# Balance between Noise and Information Flow Maximizes Set Complexity of Network Dynamics

**DOI:** 10.1371/journal.pone.0056523

**Published:** 2013-03-13

**Authors:** Tuomo Mäki-Marttunen, Juha Kesseli, Matti Nykter

**Affiliations:** 1 Department of Signal Processing, Tampere University of Technology, Tampere, Finland; 2 Department of Mathematics, Tampere University of Technology, Tampere, Finland; University of Adelaide, Australia

## Abstract

Boolean networks have been used as a discrete model for several biological systems, including metabolic and genetic regulatory networks. Due to their simplicity they offer a firm foundation for generic studies of physical systems. In this work we show, using a measure of context-dependent information, set complexity, that prior to reaching an attractor, random Boolean networks pass through a transient state characterized by high complexity. We justify this finding with a use of another measure of complexity, namely, the statistical complexity. We show that the networks can be tuned to the regime of maximal complexity by adding a suitable amount of noise to the deterministic Boolean dynamics. In fact, we show that for networks with Poisson degree distributions, all networks ranging from subcritical to slightly supercritical can be tuned with noise to reach maximal set complexity in their dynamics. For networks with a fixed number of inputs this is true for near-to-critical networks. This increase in complexity is obtained at the expense of disruption in information flow. For a large ensemble of networks showing maximal complexity, there exists a balance between noise and contracting dynamics in the state space. In networks that are close to critical the intrinsic noise required for the tuning is smaller and thus also has the smallest effect in terms of the information processing in the system. Our results suggest that the maximization of complexity near to the state transition might be a more general phenomenon in physical systems, and that noise present in a system may in fact be useful in retaining the system in a state with high information content.

## Introduction

Dynamical systems theory is being developed to understand temporal behavior of complex systems. Groundlaying studies of dynamical systems range from modeling of, e.g., genetic [1], neuronal [2], and ecological [3] networks to structural analyses of complex networks [4]–[6]. Results obtained for the function of a dynamical network of a particular type are always subject to the temporal behavior of the underlying dynamical units, which vary substantially between objects of interest [7]. To this end, Boolean network models have been used as a generic tool to study a wide range of fundamental properties of dynamical systems. These include features of attractor structure [8], information propagation and processing [9]–[11], dynamical regimes [12], structure-function relationship [13], and the ability to store information [14], [15]. Although many of these aspects can be studied with a range of other models (e.g., [16]) the strength of Boolean networks is that they are based on simple building blocks that can give rise to varied dynamics [17]. Random networks can be generated in such a way that changing one or two parameters in how the networks are generated makes the resulting network dynamics ordered, critical, or chaotic [8]. Aspects of Boolean network dynamics have been suggested as a model of biological network dynamics, such as cell types determined in part by genetic regulatory networks [18], and they have later proved efficient in, e.g., correctly reproducing observed gene expression patterns [19].

A recent development in the field of information theory is the normalized information distance [20], which can be applied to any two objects stored on a computer (e.g., genome sequences, networks, or state representations). This distance uniquely specifies the informational difference between two objects and is defined in terms of the Kolmogorov complexity. The Kolmogorov complexity [21], K(x), of an object x is defined to be the length of a shortest program to output x on a universal computer (i.e., on an all-purpose machine). Intuitively, K(x) represents the minimal amount of information required to generate x by any effective process and can be thought of as the ultimately compressed form of x. Although the normalized information distance, like the Kolmogorov complexity itself, is not computable, it can nonetheless be effectively approximated by using real-world data compressors.

Recently, a context-dependent measure of information, *set complexity*, has been applied to quantify various aspects of network topology and dynamics [22], [23]. This measure assesses the complexity of a set of strings in such a way that the approximate Kolmogorov complexities of the strings are balanced by a function of the pairwise normalized information distances within the set. The motivation for this context-dependent measure of information is that it should be able to quantify the total amount of non-redundant information, rather than the overall complexity of the data. This means that while a standard measure of information, such as Kolmogorov complexity, is maximized for random data, the set complexity quantifies the trade-off between randomness and identically repeated symbols.

The complexity of Boolean networks has hitherto been analyzed using many approaches. These include, e.g., the computational complexity of a Boolean network circuit [24], [25], the entropy of the basins of attractors [9], and the statistical complexity of the steady state of a network or the complexity of single nodes averaged over time [26]. However, the temporal complexity of the Boolean network dynamics is still poorly understood. How does the complexity of Boolean network dynamics vary in time? To what extent does the complexity change when settling to an attractor? If there are processes that allow transitions between attractors, how do they affect the complexity? Is it by any means feasible to assess the temporal complexity of Boolean networks? In our earlier study [27] we shed light on some of the questions by applying the set complexity measure to successive states of Boolean networks. We found that the complexity of the dynamics was temporally maximized near a transition to an attractor. This raised many more questions, most important ones being whether this phenomenon is real and whether the stage of maximal complexity could be prolonged by introducing noise to the network. In the present work we justify our findings using another complexity measure, namely, the statistical complexity, which was originally presented in [28] and refined in a series of papers by Shalizi [29], [30]. We also show that the high complexity can indeed be retained just by tuning the system with a suitable amount of noise. Noisy Boolean networks have been extensively studied with an aspect to robustness and stability of the attractor states [31]–[33]. In this work we employ the white noise model used in e.g. [31]. The noise imposes a challenge for the information processing and storage, and hence, we also consider the noise-induced disruption in information flow in different networks with variable levels of noise. We show that the networks near the critical regime can most easily be added a noise component that elevates the steady-state complexity value without making the dynamics too random.

## Results

### Complexity of noiseless Boolean network dynamics is temporally maximized prior to an attractor

**Figure 1:**
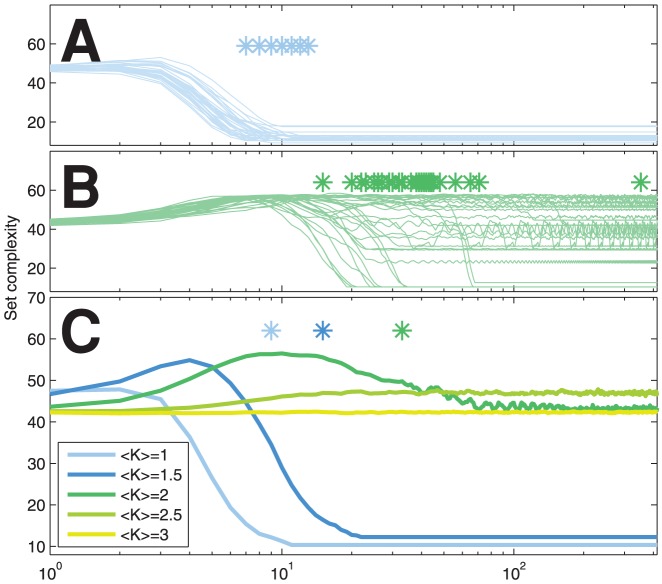
Set complexity time series for random Poisson Boolean networks shows temporal maximum prior to reaching the attractor in several networks with different mean number of inputs 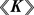
 **.** (A–B): Set complexity trajectories of single simulations of 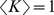 (A) and 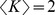 (B) networks. The first arrivals to the attractor are marked with stars. (C) The median set complexity of 100 simulation results for five different 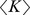
s. The stars above the curves show the median of the time instant of first arrival to the attractor.

To attain the quantification of temporal complexity, we start by reprising the study on dynamical complexity in [27], now in the context of noiseless Poisson networks. Fig. 1 shows the complexity of Poisson network dynamics as a function of time. Poisson networks with different expected number of neighbors 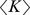 obtain statistically different dynamical complexities. As seen in [27] with fixed-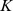 networks, Fig. 1 shows that the critical (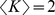) networks possess a *transient state* where the set complexity of the dynamics is maximized, and which is followed by a descent to an attractor level value. The transient state is also observed in the slightly subcritical (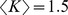) network, but not in the slightly supercritical (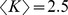) network. The dynamical complexity in highly subcritical (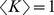) networks is quickly reduced to a steady low value that represents attaining a short cycle attractor, whereas the supercritical (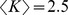, 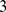) networks seldom reach an attractor by the end of the simulation. Due to long transition period the dynamics of slightly supercritical (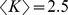) networks seem to exhibit higher steady-state complexity than critical networks (Fig. 1). This is consistent with the finite size network results reported by [11].

For reference, let us consider the extreme values for set complexity empirically. The distribution of LZMA-estimated values of 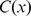, where 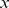 is a random binary string of length 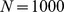, is well approximated by a Gaussian distribution with mean 224.14 and standard deviation 2.96 (data not shown) — the maximum value we came across among all data of the present work was 238. Thereby, Eq. 2 can be used to infer the maximal set complexity value for networks of this size as 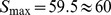, as the theoretical minimum is 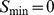. Fig. 1 shows that the range of all possible set complexity values is fairly well covered by the complexity values of RBN dynamics.

### A moderate amount of noise elevates the complexity of the network dynamics

**Figure 2:**
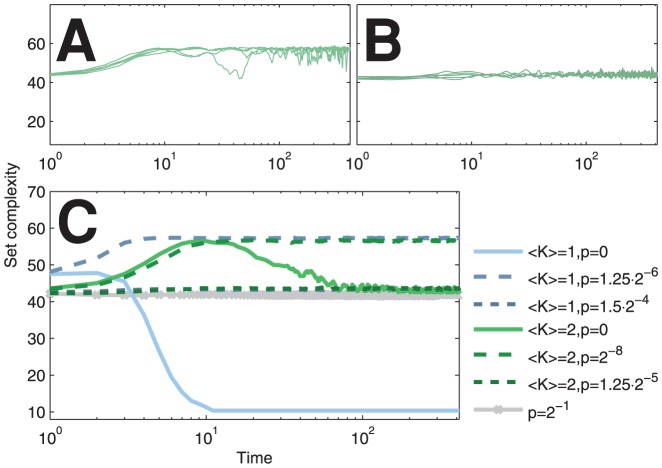
Noise can maintain the network in a high-complexity state. (A–B): Set complexity trajectories of single simulations of 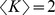 Poisson networks with moderate (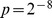, A) and high (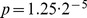, B) levels of noise. (C): Medians of set complexity trajectories for noisy Poisson networks with different degrees 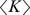 and flip probabilities 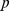. The complexity trajectory of the maximally noisy network that is identical for all 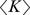 is plotted in grey. 100 independent samples were used.

To model the dynamical behavior under noisy conditions, we study the effect of nonzero flip probability 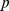. Fig. 2 shows complexity trajectories of noisy networks with zero, moderate, and high levels of noise. One can observe that for a moderate level of noise the set complexity value does not fall to a low value that is typical to a regime of noiseless ordered dynamics.

**Figure 3:**
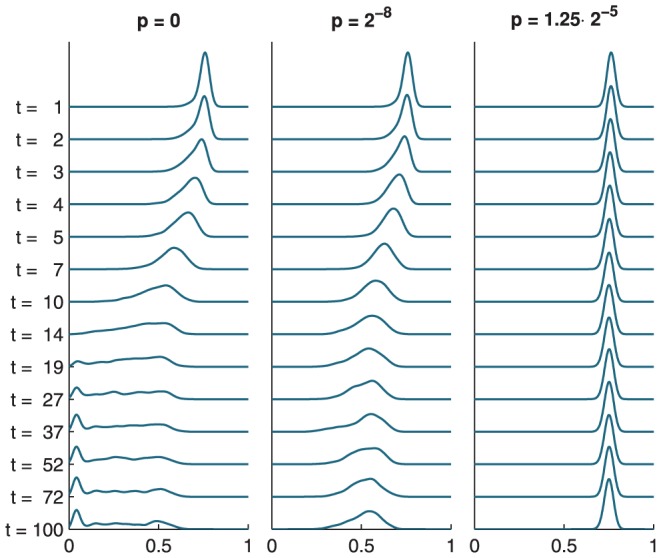
The propagation of NCD distributions explains the time course of the set complexity. The panels show the distributions of NCD values on interval 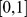 in noiseless (left), moderately noisy (middle) and highly noisy (right) Poisson networks with 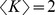. The time instant of observation grows downwards with the figures plotted: The curve plotted for 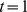 corresponds to the distribution of off-diagonal elements of NCD matrix 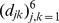, while the curve for 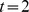 corresponds to 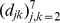, and so forth. The distributions are pooled across 100 network realizations and smoothened with a Gaussian filter with standard deviation 0.02. The mean of the NCD distribution in noiseless critical networks (left) passes 0.5 around time instant 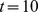, as expected from the complexity peak at 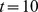 in Fig. 1. The small peaks of noiseless networks in the regime of low NCD correspond to point-attractors. In these attractors the state 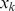 remains constant, and since the Kolmogorov complexity of a dublicated string is not much higher than that of the original (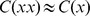), the resulting NCD values are very small. The mean of the NCD distribution in Poisson networks with moderate noise (middle) approaches 0.5 as time passes, accounting for the high set complexity values in the regime of large 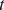 in Fig. 2. In highly noisy networks (right) the NCD distributions have only values that are notably higher than 0.5 due to the excess of randomness, and hence the low set complexity value for these networks in Fig. 2.

To explain this observation, we can analyze Eq. 2 to gain an insight into how the differences in the set complexity values arise. One can find three different causes for high values of set complexity. Firstly, the average Kolmogorov complexity 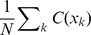 of the strings may be high, implying higher values for set complexity. Secondly, the average value of the function of NCDs (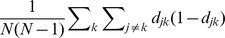) may be high, likewise increasing the set complexity. Greatest set complexities are attained when the values of NCD (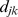) are as close as possible to 0.5, which maximizes the inline function 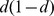. Third cause would be a combinatory effect of these two such that, although the mean values of both mentioned quantities were relatively small, there may be a few strings 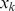 with high Kolmogorov complexity 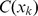 that lie on average on a distance of 0.5 from most of the other strings and hence raise the set complexity value. In Poisson networks the Kolmogorov complexities 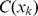 show little variation across both time and network realizations as each string is, ultimately, a random binary string with equal probabilities of 0 and 1. Therefore, the high values of set complexity must be due to the values of NCD being close to 0.5. Fig. 3 shows the evolution of the NCD distributions through time and explains the differences observed between the set complexity curves of critical networks in Fig. 2.

**Figure 4:**
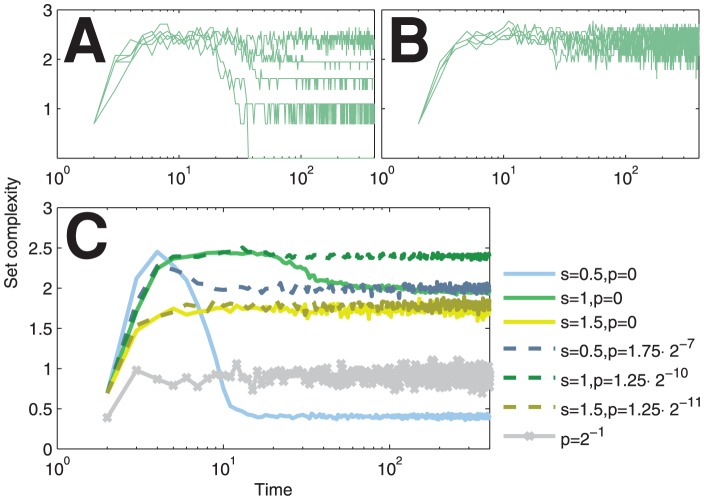
Statistical complexity produces qualitatively similar temporal complexity as the set complexity. (A–B): Statistical complexity trajectories of single simulations of noiseless (A) and noisy (B) critical networks. Both in- and out-degree of the nodes are fixed as 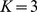. (C): Mean statistical complexity time series of subcritical (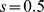), critical (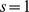) and supercritical (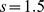) networks over 50 repetitions. The noisy networks are marked with dashed and the noiseless networks with solid line. The statistical complexity of the fully noisy (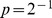) network is plotted with grey for reference.

The temporal rise and descent of the complexity in Boolean networks is not a property of the set complexity measure only. In fact, we can observe similar behavior using a measure of *statistical complexity* [30]. In this approach, the complexity is estimated as the logarithm of the number of causal states of the system. The causal states are unions of such past configurations that produce equal or almost equal distribution of the future configurations. These distributions have to be estimated from the data. The method is not as such applicable to our network types, as even the fixed-K networks have variation in the out-degree of the nodes. However, the fixed-K networks can be modified with minimal changes to produce fixed out-degree as well, and this allows the use of statistical complexity measure, yet only in the case where past and future are considered no more than one step away from the present. Fig. 4 shows the statistical complexity time series for such “fixed-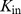-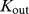
” networks.

**Figure 5:**
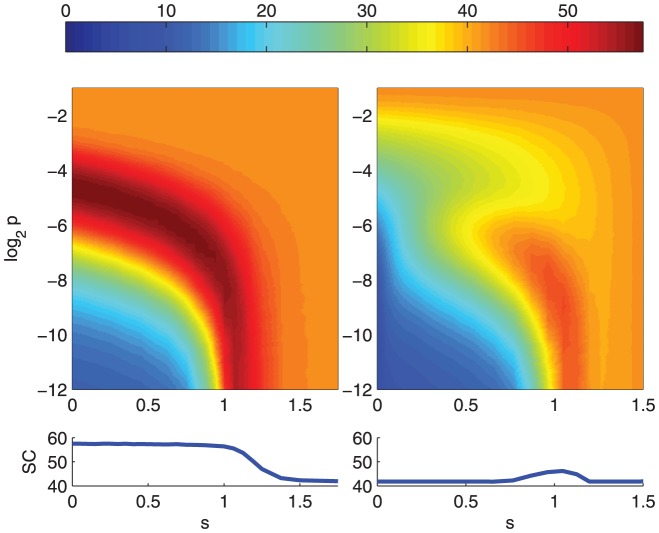
Poisson networks can be set a noise level that maximizes the steady-state set complexity. The color of the plot shows the steady-state set complexity of Boolean network dynamics for both Poisson networks (left) and fixed-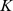 networks with 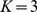 (right) as functions of sensitivity 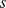 and flip probability 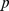. For each simulation, a median of set complexities is taken over time steps 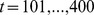. Further averaged, the color shows the median of 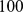 simulations, smoothened with bilinear interpolation. The lower panels show the maximum of the plane, taken over the flip probability.

Let us next quantify the difference between the networks with varying level of noise that can be observed in Fig. 2. We estimate the average set complexity of the “steady state” of the network, which we consider, in networks of this size, all but the first 100 time steps of the simulation. Fig. 5 shows the median of steady-state set complexities in Poisson networks and fixed-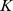 networks with 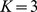. The set complexities are lowest in the regime of the most ordered dynamics (low sensitivity 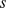, low flip probability 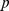). Another stable set complexity value is found in the other extreme, where the dynamics is either chaotic (large 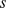) or random (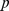 near to 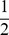), or both. Between these two extremes lies a region where the set complexity is actually higher than either of these extremes. The existence of this region is consistent for different system sizes (validated with 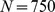 and 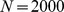, data not shown). A corresponding plot of statistical complexity in fixed-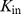-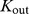 networks can be found in supplementary data (Fig. S1).

**Figure 6:**
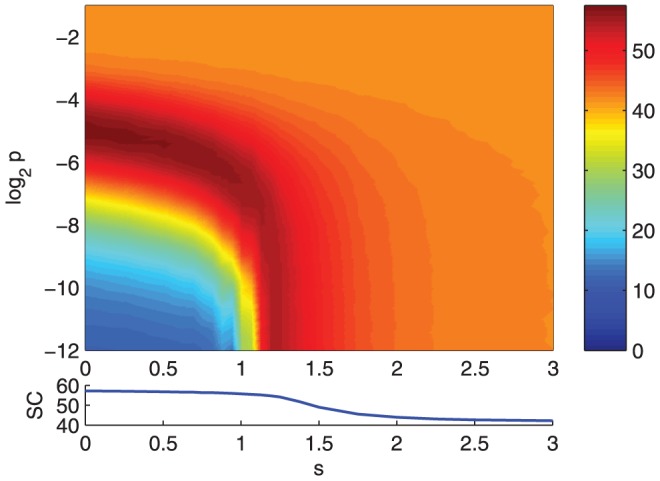
Asynchronous Poisson RBNs show qualitatively the same set complexity statistics as the synchronous ones. The color of the plot shows steady-state set complexities of asynchronous Boolean network dynamics for Poisson networks as functions of sensitivity 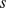 and flip probability 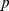. The synchronous state update described in the Methods section is replaced by 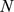 successive single-node state updates. The node to update is picked by random every time instant, and thereby after the 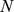 state updates some nodes have most probably been updated several times and some nodes none. The set complexities are calculated for states at the modulus-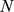 time steps 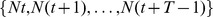. Similarly to the Fig. 5, a median of set complexities is taken over time steps 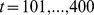, and the color of the plot shows the median of 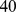 simulations, smoothened with bilinear interpolation. The lower panels show the maximum of the plane, taken over the flip probability. A slight difference to Fig. 5 is that in asynchronous networks the high-complexity regime extends more to the chaotic (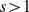) regime. This is in agreement with [46], where networks with random asynchronous updating schemes were observed to reside more often in an attractor than their synchronous counterparts, suggesting that their dynamics be on average more redundant.

We can observe that among fixed-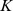 (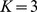) networks the ones near the critical network, which by Eqn. 1 is obtained by choosing the bias as 
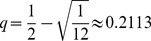
, produce the maximal steady-state complexity. One can also observe that among Poisson networks one always finds a suitable noise level to obtain a near-to-maximal steady-state complexity (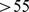) as long as the sensitivity is restricted (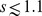). Qualitatively the same result can be obtained with *asynchronous* random Boolean networks, as Fig. 6 shows.

**Figure 7:**
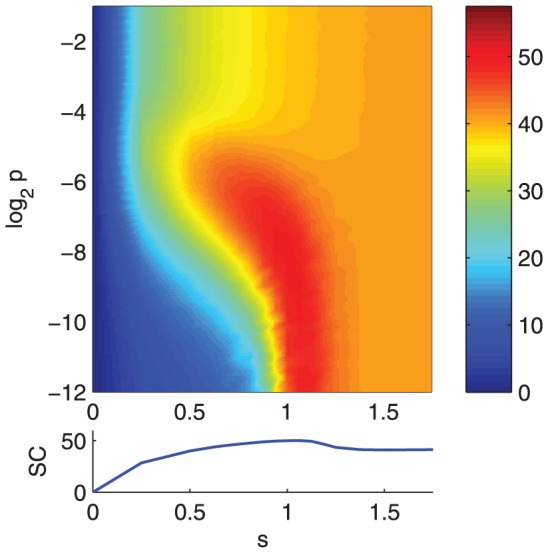
The subcritical Poisson networks lose their high steady-state complexity when nodes with zero inputs are neglected. In this figure, the set complexity is calculated similarly to the Poisson network steady-state complexity in 5, but only states of those nodes that receive at least one input from the system are included in the strings 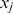.

What is rather non-intuitive about Figs. 5 and 6 is the high complexity of noisy low-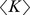 Poisson networks, where a large proportion of the nodes receive zero inputs. The dynamics of these nodes are purely an effect of the noise that occasionally pushes the nodes from their constant output. The effect they have on the set complexity values of the dynamics is twofold. Firstly, from the temporal aspect these nodes lie somewhere between chaos and order, as they most of the time have constant value but may change their value temporarily. Secondly, although the surrounding nodes do not affect the dynamics of these nodes, these nodes might still output to other nodes, and hence the noisy nature of these nodes may contribute to the rest of the system. Clearly, we would like to diminish the first-mentioned effect without removing the latter aspect. Therefore, we repeat the set complexity calculation of Fig. 5, but neglect the nodes that we know to receive no input from the system. In other words, the dynamics of the system remains untouched, but the complexity is calculated only over those nodes that receive one or more inputs. Fig. 7 shows the steady state set complexity values of such networks. One can observe that the set complexity value for networks approaches zero as 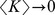, which is due to the ever shortening strings 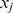 — and ever diminishing Kolmogorov complexity 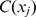. What remains unchanged from Fig. 5 is the high complexity of networks near to criticality, where the critical and subcritical networks have to be tuned with moderate level of noise in order to obtain the maximal complexity and the slightly supercritical networks attain it with no or little noise. One should note that in order to perform the complexity analysis in this way we need external information on the network structure, in the minimum the notion on which nodes do not have any inputs. By contrast, when we assess the set complexity of the dynamics using all available nodes, no information on the structure of the network is required.

How great are these mentioned “moderate” levels of noise? In critical 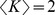 Poisson networks the maximal steady-state set complexity was attained with flip probability 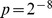, while in subcritical 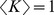 and 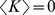 networks it is attained with 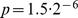 and 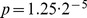, respectively (Fig. 5). In the system size 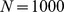 these levels of noise mean that in the subcritical networks on average 39 (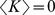) or 23 (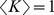) nodes are flipped every time step, and in the critical network on average 3.9 nodes. In the critical network also much smaller noise levels suffice to attain 95% of the overall maximal steady-state complexity (the least noise level for this is 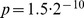, i.e., the states of 1.5 nodes on average flipped every time step). The same cannot be said of 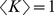 and 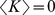 networks, which attain the 95% of the overall maximum set complexity at the noise levels of 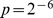 and 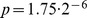, respectively.

**Figure 8:**
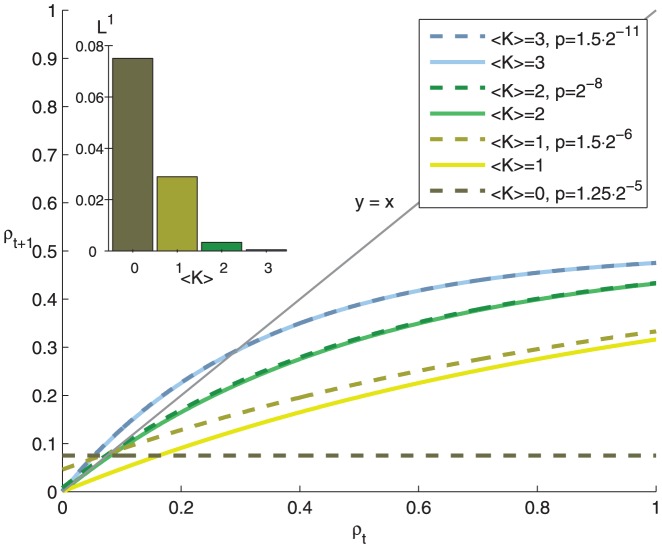
Subcritical networks with maximal steady-state set complexity suffer from disruption in information flow. The figure shows the Derrida curves of different networks according to Eq. 4. The networks are Poisson networks with 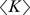 = 0,1,2,3, where for each network the noise level is chosen such that the steady-state set complexity is maximized (dashed lines), and the corresponding noiseless networks (solid lines). The noiseless 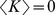 network is not plotted, as it has the property that 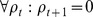. The thin grey line shows the diagonal 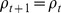, which would correspond to the state-preserving network 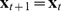. **Inset**: The 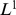 norm between the noisy and the corresponding noiseless networks.

The contribution of different levels of noise to the Boolean network dynamics can also be characterized by their Derrida curves (Eqn. 4). These are plotted for Poisson networks with 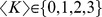 in Fig. 8. For each network both noiseless and noisy case are plotted, where the noise level is chosen as the one that produces the maximal set complexity in Fig. 5. The critical and chaotic (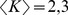) networks with noise are very similar to the corresponding noiseless (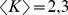) networks in Derrida sense, whereas the noisy subcritical networks (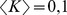) show greater difference from the corresponding noiseless networks. The inset in Fig. 8 shows the 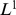 difference between the noisy and noiseless curve for each 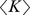. This value represents the average amount of perturbation that is due to the noise, and can be considered the *perturbation-averaged disruption in information flow* of the system. For instance, the 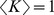 network with the noise level that produces maximal complexity adds on average 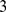 percentage points to the perturbation of the noiseless network, while the corresponding values for 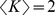 and 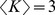 network are 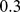 and 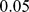 percentage points, respectively. This suggests that the chosen level of noise for subcritical networks is too great for the network to maintain the meaningful information in their dynamics.

## Discussion

In this work we have shown that the steady-state complexity in Boolean network models can be maximized by choosing the noise level appropriately. In fixed-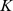 networks with 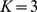 this is plausible only for near-to-critical networks (Fig. 5, S1). For Poisson networks this is possible for both sub-critical and near-to-critical networks (Fig. 2, 5). However, the levels of noise that maximize the set complexity in subcritical Poisson networks imply large decrease in information flow compared to those near criticality (Fig. 8). In addition, neglecting the nodes to which the system does not contribute fades the high complexity of these subcritical networks (Fig. 7). The results shown are qualitatively robust to changes in system size 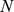, and the main result is confirmed with asynchronous Boolean networks (Fig. 6).

The complexity of dynamics is in this work primarily assessed through a measure of context-dependent information, i.e., set complexity [22], of successive states of the network. While a measure of context-independent information (such as Kolmogorov complexity) would increase with the unpredictability of the states, that is with the flip probability, the context-dependent information starts to decrease after reaching a certain level of noise (Fig. 5). We have oserved a similar result for the saturation and descent of set complexity in the context of a lattice gas system [27]. The shown results suggest that maximization of the complexity at the edge of chaos and order is robust to the choice of paradigm: One finds it either by adding order into chaotical dynamics, as is the case when a random Boolean network state approaches a short-cycle attractor (Fig. 1), or by increasing randomness into a system with ordered dynamics, as shown with the steady-state complexities of noisy Boolean networks (Fig. 5).

The fact that the complexity measure is maximized at the edge of chaos and order (and not in the totally unpredictable regime as is the case with Kolmogorov complexity) is not characteristic of the set-based complexity measure only, but is a design principle for many other measures of complexity [34], [28], [35], [36]. The common trend for complexity measures — stated even as a requirement for complexity measure in [34] — is that they are based on entropy or Shannon information, and are consequently dependent on the underlying prior distribution of the strings whose complexity is to be assessed. This prior knowledge is rarely at hand in, for instance, applications of biology, as discussed in [22]. For reference, we confirmed the main result with one such measure applicable to time series data, namely, the statistical complexity [30], where the state distributions are estimated from the data (Fig. 4, S1). The presented method of estimating the statistical complexity requires a fixed number of inputs and outputs for each node, and hence it could not be applied to Poisson networks, nor to fixed-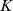 networks without modifications. In addition, the structure of the network must be known in order to estimate the statistical complexity. By contrast, the measure of set complexity is very flexible and does not require any knowledge on the state distributions nor the network structure. On the other hand, the set complexity is based on the Kolmogorov complexity, which has shown to be uncomputable in general. To this end, the use of general data compression algorithms for aprroximation of Kolmogorov complexity has proven to be a powerful tool. As an example, phylogenetic trees and language family trees have been successfully reconstructed in [37] and [20] using methods that approximate Kolmogorov complexity with data compressors. In [20], the reconstruction is based on NCD estimated using several different data compressors, as the authors of [37] utilize only Lempel-Ziv algorithm for estimating the Kolmogorov complexity but several similarity metrics closely related to NCD. Built upon NCD, there is a great promise also in the set complexity measure. Although it was originally proposed as a heuristic measure, the set complexity has since then been shown to possess optimal properties in, e.g., assessing the structure of complete bipartite graphs [38].

The states with maximal complexity are of interest for several reasons. As discussed in [22] with aspect to biological systems, a high value of set complexity reflects large amount of meaningful information. In our earlier work [27] and in Fig. 1 we have shown that the temporal context-dependent information content in noiseless systems is maximized prior to reaching the attractor. This could mean that the system, if interpreted as a “decision maker” of on which attractor to fall, performs the crucial decision during this stage and not earlier when the dynamics are of low information content due to the lack of context, nor later when the dynamics are redundant. The interesting result reported in the present paper is the effect of moderate level of noise on the elevated steady-state complexity of the system. This suggests that a moderate level of noise be helpful in retaining the system in an agile state, i.e., ready to act in a meaningful way to different cues.

Our finding that asynchronous and synchronous random Boolean networks have very similar steady-state complexity behavior (Fig. 5 and Fig. 6) is a rather surprising result. Earlier theoretical and computational analyses show grave differences between these two model classes in, e.g., number of attractors [39] and Derrida curves [40]. However, both of these aspects may suffer from comparing the uncomparable. For instance, in synchronous RBNs attractors can be either point or cyclic attractors, as in asynchronous RBNs they are either point or loose attractors. As for the Derrida-based analysis, the ways to define the Derrida curve for asynchronous Boolean networks are many. The authors of [40] choose to compare the two runs after one synchronous update of a number 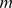 of nodes (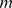 picked from a uniform distribution from 1 to 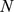), while it might be more relevant to make the comparison after 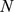 updates of single node. By contrast, our analysis, which is based on the amount of redundancy in the steady-state dynamics, does not require a definition of any intermediate parameter of the dynamics, but is straightforwardly applicable to any discrete-time discrete-state system. Ultimately, assessing the set complexity of the steady-state dynamics could form a novel, intricate way of characterizing complex networks.

In addition to models of Boolean networks, above analysis is highly relevant also for understanding more complex dynamical systems. Living cells for example, need to maintain their homeostatic state under noisy environment. Early studies with Boolean networks have addressed the question of homeostasis by studying the effect of small perturbations [41]. We have shown how the Boolean network model parameters together with noise control information flow in the system. Our analysis in Fig. 8 shows that if too much noise is added to gain higher complexity, the system can no longer maintain its dynamical function. This is a hallmark event of the loss of homeostasis. The presented framework could serve as a general basis for estimating the noise levels that a given system can tolerate and still maintain its dynamical function, or a homeostatic state.

We have studied information flow in systems without external stimuli, but an important and much more challenging question is the homeostasis in systems that receive and transfer information. This could correspond to the case where a system is not only retaining current state information under noise but is also trying to adapt and respond to systematic changes in the surroundings. In doing this, the task is to filter useful information from the external signals, which also include noise. A key question for future studies is to analyze the connection between external and internal information and noise in the system. The real signicance of maximal complexity states could be in having suitable versatility to perform the filtering task efficiently, and tuning the system by noise may help in such filtering tasks as well.

## Materials and Methods

### Boolean networks

A *synchronous Boolean network* is defined as a collection of nodes 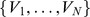 where at each time step 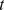 each node is assigned a Boolean value 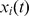, i.e.
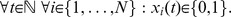
Here, 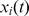 is the state of the node 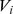 at time instant 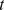. Each node receives input from 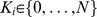 nodes and the state of the node at time instant 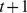 is a Boolean function of the states of its input nodes at time instant 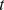:
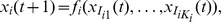
where 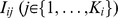 are the indices of the input nodes of node 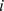.

In this work we use two types of synchronous random Boolean networks. The first class of networks is such that the number of inputs to a node is picked from a Poisson distribution and the input nodes are picked by random, creating a Poisson distribution for the outputs of the nodes as well. The update functions 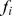 are also picked by random, i.e., each combination of inputs is assigned an output value 0 or 1 with equal probabilities 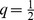. We refer to these networks as *Poisson networks*. In the other class of networks the number of input nodes is fixed. In this class functionally different networks are obtained by changing the probability 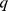 (also called the bias of the network) of Boolean function output being 0. We call this class of networks *fixed-* 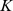 *networks*. The dynamics in both Poisson and fixed-K networks can be characterized by *sensitivity* 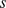, which is calculated [42] as
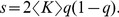
(1)Networks with 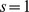 are considered critical, as lower and higher sensitivity values correspond to subcritical and supercritical dynamics, respectively [13]. Both types of networks can be assigned a level of noise through a nonzero flip probability 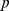: At each time step for each node, there is a probability of 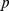 of getting the opposite state than the one dictated by the deterministic dynamics.

We consider networks of size 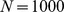 with variable levels of noise. The complexity of network dynamics at time 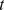 is estimated using the set complexity over 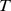 successive states: 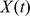, 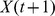, …, 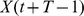. The value of 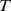 used in the calculation determines the time resolution obtained, and has to be selected to correspond with the transient lengths observed. The results are consistent for 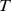 ranging from 2 to 10 — in this work, we present results for 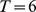. The complexity of dynamics is assigned for time instants 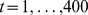. The initial state of the network is picked by random from a uniform distribution over the state space.

### NCD and set complexity

We study the complexity of Boolean network dynamics following the framework we presented in [27]. The dynamics of a Boolean network is represented by a set of its successive states that are read into strings. To the obtained set of strings one applies the *set complexity* measure [22], defined as:
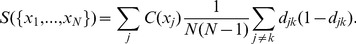
(2)The function 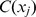 denotes the approximation of Kolmogorov complexity of string 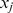. The variable 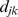 represents the *normalized compression distance* (NCD), a computable approximation of the normalized information distance [20] of strings 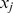 and 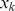, defined as
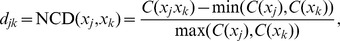
where 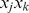 is the concatenation of strings 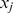 and 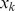. 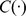 is calculated using LZMA compression.

For the basic properties of the set complexity measure we refer to [22], which shows, e.g., the effect of increasing level of noise on the resulting set complexity value of identical strings. In this work, we use the set complexity exclusively for time series data. We therefore illustrate the behavior of the set complexity in the case of random, periodic and quasiperiodic dynamics in the supplementary data of this paper (Fig. S2). These three types of dynamics are relevant in our study, as the Boolean network dynamics is *periodic* in the noiseless case (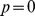) and *random* in the case of maximal noise (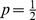). The dynamics in the case of moderate noise levels could be viewed as *quasiperiodic*. In the example of Fig. S2, the periodic dynamics produces the least set complexity values, while the complexity of the quasiperiodic dynamics is on average higher than either the periodic or random dynamics.

Generally, the framework of NCD allows the use of any lossless data compression method for the estimation of Kolmogorov complexity. However, in order to obtain reliable results the most efficient — in terms of compression ratio — should be used when possible. We have reviewed the use of different compressors for estimating Kolmogorov complexity in our earlier work [16], [43]. In [43] the LZMA algorithm was found most efficient in compressing long repeated strings. In [43] an adaptive packing algorithm called prediction by partial matching (PPM) [44] was found in some aspects superior to LZMA. However, PPM produced in many cases NCD values larger than 1, which is not allowed when computing the set complexity. We have not encountered such problems with the use of LZMA algorithm. The LZMA software used in this study is LZMA SDK 4.65.

### Statistical complexity

The statistical complexity [29], [30] is defined as the amount of information in the statistic that is minimal and sufficient for predicting the future of the process. This is done locally through parametrization of the past states of the nodes that could affect the state at hand, and similarly, the future states that the node at hand could affect. These past states are referred to as the *past light cone* and the future states as the *future light cone* — the past light cone includes the state of the considered node at the present time step [29]. The objects of interest are the conditional distributions 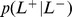, where 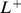 and 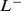 are the future and past light cones, respectively. Whenever two past light cones produce the same distribution of future light cones, these past light cones are considered to belong to the same *causal state*. The statistical complexity of the process at time 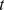 is calculated as the logarithm of the number of causal states at that moment.

We follow the example given in [30]: We consider only one step into the past and into the future and estimate the number of causal states. To do this, we repeat each network simulation 50 times from random initial state in order to estimate the conditional distributions 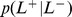 at each time step and apply Pearson's 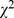-test with p-value 0.05 to obtain the causal states from them. Both in- and out-degree of the nodes are fixed to 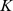 in order to make the comparison of distributions possible. Our method is, however, different in one aspect. We quantify the states *relative* to the past state. That is, we consider the state of node 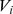 as 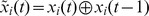 instead of the absolute state 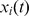, where 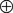 represents the exclusive or (XOR). This choice is due to the random choice of the Boolean functions: As we consider only one step ahead, we can only expect the absolute future states to be distributed as repeated Bernoulli distribution 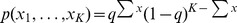, while the distribution of the relative states successfully captures the dynamics of the system.

### Derrida curves for Poisson networks

Derrida analysis [42] is a widely-used method for studying the dynamical behavior of discrete systems. The Derrida curve shows the average difference between the states of two identical networks at time instant 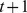 given their difference at time instant 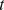. To compute this curve we consider a noisy Poisson Boolean network, initially at state 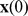, and a perturbed run of the same network, initially at state 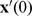. The state update can be decomposed to two discrete stages. The first stage (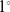) is the deterministic update 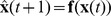, and the second stage (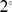) is the possible bit flip, defined as
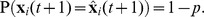
The possible bit flips in the two runs occur independently of each other. Let us denote the fraction of nodes whose states are different in the two runs by 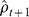 (after 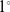) and 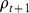 (after 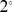).

For simplicity, we consider networks in the limit of the system size 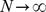. The number of inputs to a node in the network is distributed as Poisson: 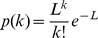, 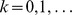, where 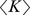 is represented by 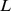 for the sake of clarity. By the randomness in the choice of function 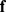, the probability of a node in the perturbed run having a different value from the one in the reference run after 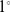 is
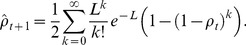
(3)In the stage 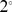 there is a probability of 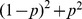 that the states of the two runs stay the same with respect to one another, and a probability of 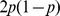 that exactly one of the two bits is inverted. Hence we have
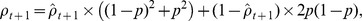
which with Eq. (3) and a bit of algebra gives
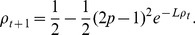
(4)Note that we applied the assumption of independence of the state 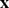 and the random function 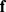 in the derivation of Eq. (3). This assumption is fully valid only during the first state update 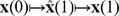. However, Derrida and Weisbuch [45] among others have shown that this *annealed approximation* predicts many aspects of network dynamical behavior to a fine degree.

## Supporting Information

Figure S1 **Networks near to critical can be tuned to maximal statistical complexity.** The color of the plot shows the steady-state statistical complexity of fixed-K_in_-K_out_ networks as function of sensitivity *s* and flip probability *p*. See Fig. 4 for reference. The result shown is the median of 65 network repetitions, smoothened with bilinear interpolation. The lower panel shows the maximum of the steady-state statistical complexity over the flip probability *p*.
(EPS)

Figure S2
**Set complexity time series for random, periodic and quasiperiodπic dynamics.** The upper panels show the control signals, which are functions 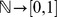 that have either random (left), periodic (middle) or quasiperiodic (right) behavior. The periodic signal is chosen as 
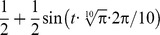
, where 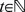. The factor 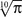 is added to ensure that the control signal does not receive an exactly same value at distinct time points in finite time. The quasiperiodic signal is chosen as an interpolation of two periodic signals as 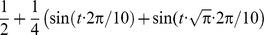. In each of the three cases a set of 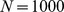 nodes are created, and each node is given a random threshold between 0 and 1. When the control signal is above the threshold, the node output is 1, and 0 otherwise. The middle row panels show the dynamics of the nodes, black representing 1 s and white 0 s. In the lower panels the curves show the average values (100 repetitions) of the set complexity trajectories of these systems. The set complexity is calculated using 20 successive time steps. The values of set complexity are lowest for the periodic system, and second lowest for the random data, while the quasiperiodic system produces the highest average set complexity.(EPS)
